# Short-term efficacy and safety of A-stream glaucoma shunt: a 6-month study

**DOI:** 10.1038/s41433-025-03728-y

**Published:** 2025-02-21

**Authors:** Hae Min Park, Eun Jung Lee, Jong Chul Han, Seungsoo Rho, Jong Hoon Shin, Do Young Park

**Affiliations:** 1 Department of Ophthalmology, Samsung Medical Center, Sungkyunkwan University School of Medicine, Seoul, Republic of Korea; 2 Department of Ophthalmology, CHA Bundang Medical Center, CHA University, Seongnam-si, Republic of Korea; 3 PNU Ophthalmology Clinic, Busan, Republic of Korea

**Keywords:** Glaucoma, Implants

## Abstract

**Purpose:**

This study evaluated the short-term efficacy and safety of the A-stream Glaucoma Shunt (A-stream; MICROT Inc., Republic of Korea) in patients who completed 6 months of follow-up after implantation.

**Methods:**

Medical records of 49 patients (49 eyes) who underwent A-stream implantation between October 2023 and February 2024 were retrospectively reviewed. Primary outcomes included surgical success and intraocular pressure (IOP) reduction at 6 months postoperatively. Success was defined as achieving an IOP ≤ 18 mmHg, with at least a 20% reduction from preoperative IOP, and without clinically significant hypotony (IOP < 6 mmHg persisting >1 month or with hypotony maculopathy), classified as qualified (with or without medications) or complete (without medications). Ripcord removal timing and its effect on IOP, postoperative interventions, and complications were analysed.

**Results:**

At 6 months, the mean IOP significantly decreased from 26.9 ± 8.3 mmHg to 11.9 ± 3.5 mmHg (*P* < 0.01). Complete success was achieved in 77.6% and qualified success in 93.9% of the eyes. The ripcord was removed in 73.5% of eyes at an average of 1.8 ± 1.3 months postoperatively, which led to further IOP reduction of 6.8 ± 5.0 mmHg 1 month after removal. No cases of clinically significant hypotony or vision-threatening complications were observed.

**Conclusions:**

The A-stream demonstrated excellent short-term efficacy and safety in lowering IOP with high success rates. The ripcord enabled controlled IOP adjustments postoperatively. Further studies are warranted to evaluate long-term outcomes and compare them with conventional surgeries.

## Introduction

Glaucoma is a leading cause of irreversible blindness worldwide and is predominantly managed by lowering intraocular pressure (IOP) [1]. Surgical intervention is crucial for patients who are unresponsive to medications or laser treatments. Trabeculectomy (TRAB) has been the gold standard for treatment since the 1960s. However, TRAB poses significant risks including unpredictable outcomes, long learning curves for surgeons, and sight-threatening complications [2–4].

Minimally invasive glaucoma surgery (MIGS) aims to mitigate these complications and is a safer alternative to TRAB for achieving target IOP levels. Despite advancements, traditional MIGS devices, which primarily targets the trabecular meshwork, face limitations in efficacy owing to pre-existing resistance in the collector and episcleral venous systems [5, 6].

Recently, new subconjunctival microfiltering devices such as the PreserFlo Microshunt (PreserFlo) (Santen Pharmaceutical Company Ltd., Osaka, Japan) and XEN 45 gel stent (XEN) (Allergan, AbbVie Company, Dublin, Ireland), have been developed. These devices utilise similar outflow pathways as TRAB by creating a filtering bleb, but unlike TRAB, they enable consistent aqueous humour outflow from the anterior chamber to the filtering bleb via a tube. Therefore, these devices are recognised for balancing the efficacy, safety, and procedural simplicity between TRAB and traditional MIGS [7, 8]. However, in cases when IOP rises after the initial postoperative period, these devices, such as XEN or PreserFlo, lack a direct equivalent to the suture lysis procedure used in TRAB to achieve additional IOP reduction. This limitation could potentially result in less favourable long-term outcomes compared to TRAB in which adjustments can be made to obtain optimal IOP levels postoperatively [9–12].

**Fig. 1:**
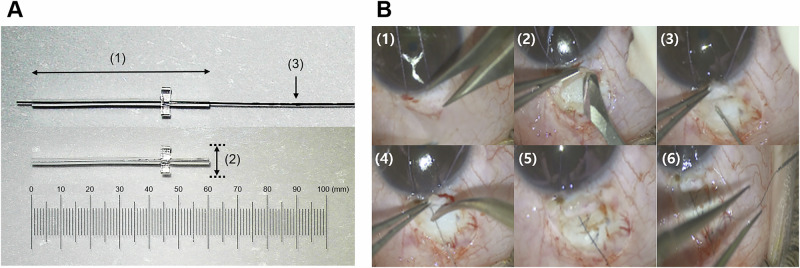
A-stream glaucoma shunt and surgical procedure. **A** Structural features and design. The A-stream glaucoma shunt is shown with the ripcord (above) and without the ripcord (below). The device is made of medical-grade silicone, measuring 6 mm in length (1) with a 1-mm wing plate (2) for stable positioning. The luminal diameter is 100 μm. A pre-placed 7-0 nylon ripcord (3) is incorporated to allow postoperative adjustments. **B** Surgical procedure for A-stream implantation. (1) Fornix-based conjunctival peritomy. (2) Formation of a 3 × 3 mm scleral flap, followed by mitomycin-C application. (3) Scleral puncture using a 30-gauge needle to create an entry into the anterior chamber. (4) A-stream insertion into the anterior chamber and precise positioning. (5) Suturing of the A-stream and ripcord onto the sclera. (6) Burying the ripcord under the conjunctiva using a 30-gauge needle, followed by conjunctival suturing.

The A-stream Glaucoma Shunt (A-stream) (MICROT Inc., Seoul, Republic of Korea) represents a novel glaucoma tube shunt recently approved for use in the Republic of Korea. It was designed to achieve the effectiveness of TRAB while enhancing postoperative IOP predictability and procedural simplicity. This glaucoma shunt device features a 6 mm length and 100 µm lumen diameter silicone tube with pre-placed ripcord made from commercially available 7-0 nylon, allowing for adjustable flow control (Fig. 1A). This design aims to reduce early hypotony and allow stepwise postoperative IOP control through ripcord retraction or removal. However, comprehensive clinical data on the commercially available A-stream are required.

This study, for the first time, evaluated the short-term outcomes of A-stream up to 6 months post-surgery and focused on its effectiveness in IOP reduction, stepwise IOP control using the ripcord, and safety profile.

## Methods

### Study design

This multicentre, retrospective study included consecutive patients who underwent A-stream implantation between October 2023 and February 2024. All operative procedures were performed by fellowship-trained glaucoma specialists, following a standardised surgical protocol.

The study was conducted in accordance with the principles of the Declaration of Helsinki. Institutional review board (IRB) approval was obtained from the Samsung Medical Center (#2024-08-010) and CHA Bundang Medical Center (#2024-08-035). The IRB waived the requirement for informed consent due to the retrospective nature of the study and the absence of potential harm to participants.

### Inclusion and exclusion criteria

Consecutive eyes that underwent A-stream implantation between October 2023 and February 2024 with at least a 6-month follow-up after surgery were included. Patients diagnosed with primary open-angle glaucoma (POAG) or secondary glaucoma, including pigmentary, pseudoexfoliation, uveitic, steroid-induced, and mixed mechanism, were included regardless of glaucoma stage. Patients with a history of simple cataract surgery, laser treatment, or single TRAB, in whom sufficient uninvolved conjunctiva remained for filtering surgery, were also included.

The A-stream was implanted in eyes in which IOP exceeded the target level or where glaucoma progression was evident despite maximally tolerated medical therapy. Surgery was performed based on clinical judgement following the criteria typically used for TRAB. During the study period, no other filtering surgeries, such as TRAB or XEN gel stent implantation, were performed, and all patients underwent A-stream implantation to maintain consistency in the surgical approach.

Eyes with a history of vitrectomy or diagnoses of closed-angle, juvenile open-angle, congenital, or neovascular glaucoma were excluded from the analysis.

### Baseline characteristics

Baseline characteristics including age, sex, the affected eye, diabetes status, and hypertension were recorded. Ocular characteristics such as IOP at the time of decision for surgery (decision IOP), preoperative number of glaucoma medications, glaucoma diagnosis, visual field mean deviation (MD), histories of previous cataract surgery, other ocular surgeries, and laser trabeculoplasty were also obtained.

### Outcomes

The primary outcome was surgical success rate at the 6-month follow-up. Surgical success was defined using two criteria: achieving an IOP ≤ 18 mmHg without clinically significant hypotony and at least a 20% reduction from the decision IOP. Surgical success was further categorised as complete success, defined as achievement of the success criteria without the use of glaucoma medications, and qualified success, defined as achievement of the success criteria with or without the use of glaucoma medications. Temporary use of glaucoma medications during periods of transient IOP elevation, which were discontinued after ripcord removal, was not considered a failure. All IOP measurements were obtained using Goldmann applanation tonometry.

Hypotony was defined as an IOP of <6 mmHg. Transient hypotony refers to cases in which IOP < 6 mmHg did not persist beyond 1 month and was not associated with hypotony maculopathy. Clinically significant hypotony was defined as an IOP < 6 mmHg persisting for >1 month or associated with hypotony maculopathy. Immediate failures were defined as the need for surgical intervention or experiencing vision-threatening complications.

In addition, the surgical success rate was evaluated using an alternative upper IOP threshold of 21 mmHg. Postoperative IOP and number of medications were recorded at 1 day, 1 week, and 1, 3, and 6 months. The timing of ripcord removal and the number of cases were documented, and subsequent IOP changes were assessed, including the IOP-lowering effect 1 month after ripcord removal.

### Complications

Postoperative interventions and complications were recorded. Complications included hypotony maculopathy, bleb leaks, choroidal detachment, hyphaema, and implant exposure. Vision-threatening complications included vitreous haemorrhage, retinal detachments, suprachoroidal haemorrhage, malignant glaucoma, endophthalmitis or blebitis, corneal decompensation, and vision loss without light perception. Any revisions and reoperations were also documented.

### Device design

The A-stream is a silicone-tube glaucoma shunt device with a ripcord stent implanted via an ab externo approach to create a conjunctival filtering bleb. The A-stream received approval from the Ministry of Food and Drug Safety and was registered for use in the surgical treatment of patients with glaucoma, which highlights its readiness for clinical application in the Republic of Korea.

In detail, the A-stream design features a silicone tube with a length of 6 mm and a lumen diameter of 100 µm, stented with a 7-0 nylon ripcord pre-placed inside the tube (Fig. 1A). It ensures predictable reduction in IOP while preventing early postoperative hypotony and broadening of the intraluminal space via ripcord removal that allows for stepwise reduction during the postoperative period. Such distinguishable device features had been employed based on previous experimental studies that tested various tube diameters, both with and without the ripcord, and their effects on postoperative IOP [13, 14]. Apart from the tube diameter, a set of wings located asymmetrically near one end of the tube facilitates correct orientation and secures stable device attachment to the sclera. The A-stream is constructed from medical-grade silicone, which is soft and flexible, enabling ease of handling during implantation and reducing the likelihood of exposure by conforming to the natural curvature of the eye.

### Surgical procedure

Figure 1B illustrates the surgical steps involved in the A-stream implantation. The key steps of the surgical procedure were consistently applied across all sites and surgeons to minimise variability and ensure consistency in the surgical approach. All surgical procedures were performed under retrobulbar or sub-Tenon anaesthesia. In eyes with a history of failed glaucoma filtering surgery, the A-stream was implanted in the contralateral conjunctiva to avoid the previous surgical site. A traction suture was placed on the superior cornea and 1% lidocaine was injected into the sub-Tenon space. Conjunctival peritomy was performed, followed by the dissection of Tenon’s capsule. Haemostasis was achieved using diathermy to maintain a clear surgical field. Mitomycin-C (MMC) at a concentration of 0.4 mg/mL was applied to the bare sclera for 3–5 min using surgical sponges soaked in MMC, followed by gentle irrigation with balanced salt solution. The application time was determined by the surgeon based on patient-specific factors such as surgical history and the conjunctival condition. This protocol mirrors that used in TRAB and was consistently followed by all surgeons. A 3 × 3 mm scleral flap was created. Using a 30 G needle, a sclerostomy was made at the surgical limbus, and the A-stream was inserted into the anterior chamber. The tube tip (~1.5–2.0 mm) was placed in the anterior chamber, with 4.0 mm of the tube positioned under the scleral flap. To ensure stability, the A-stream was sutured to the sclera with 10-0 nylon both anterior and posterior to the wings. Particular attention was paid to position the wings of the A-stream under the scleral flap to minimise direct contact with the overlying conjunctiva or Tenon’s capsule. The anterior chamber portion of the implant with the ripcord was visually checked to ensure that it was not too close to the iris or corneal endothelium. Flow through the A-stream was confirmed by the presence of a drop at the end of the tube. The ripcord was tightly sutured to the sclera using 10-0 nylon to prevent unintentional removal. Finally, the conjunctiva was sutured watertight using 10-0 nylon. The ripcord was partially buried under the conjunctiva using a 30G needle to reduce irritation.

### Ripcord removal

The criteria for ripcord removal were based on IOP, morphology and characteristics of the filtering bleb, and the number of months after surgery. The ripcord was generally removed when IOP exceeded 16 mmHg at an average of two months after surgery, as IOP elevation typically occurs between the second and third postoperative months. In cases where the postoperative IOP exceeded 16 mmHg within the first 2 months, early removal was considered if features suggestive of a failing filtering bleb—such as encapsulation, flattened bleb height, or hypervascularization—were present. However, ripcord removal was not attempted within the first postoperative month because of the risk of severe hypotony. During the early period, IOP was managed with glaucoma medications. Removal within the second month was considered cautiously, balancing IOP levels and bleb morphology against the risk of hypotony. The IOP was measured 30 min after ripcord removal. In cases where IOP in the low teens persisted beyond 2–3 months, the ripcord was retained for an extended period, particularly when the filtering bleb demonstrated a diffuse and well-formed morphology.

### Postoperative management

The patients were instructed to discontinue all glaucoma medications. Topical prednisolone acetate 1% (8 times per day) and moxifloxacin 0.5% (4 times per day) were started on postoperative day 1. Postoperative eyedrops were discontinued or tapered at the discretion of the surgeon. Patients were routinely followed up on postoperative day 1 and week 1, then at intervals of 2–3 weeks during the early postoperative period. Starting at 2–3 months after surgery, the follow-up interval was extended to 1–2 months at the physician’s discretion. During the follow-up period, glaucoma medications were temporarily prescribed if IOP exceeded the target level but ripcord removal was deemed too early (e.g., within the first 2 months postoperatively). After ripcord removal, medications were discontinued if the IOP decreased to the target range. If the IOP remained above the target level after ripcord removal, glaucoma medications were reintroduced and incrementally adjusted during subsequent visits to achieve optimal IOP control. The IOP and the number of medications use were recorded at each visit to monitor the effectiveness of these adjustments. In cases where IOP elevation persisted after ripcord removal, needling was attempted as necessary to achieve additional IOP reduction. At each visit, full anterior segment and bleb examinations were performed, along with IOP measurements. Clinical signs of possible postoperative complications such as hypotony, bleb leak, or choroidal detachment were carefully evaluated.

### Statistical analyses

Statistical analyses were performed using SPSS software (22.0 version; IBM Corp., Armonk, NY, USA). Quantitative variables were demonstrated as mean ± standard deviation, and categorical variables were summarised as counts and percentages. All statistical data were tested for normality and confirmed to meet normal distribution prior to analysis. Variance was confirmed to be similar between the groups being compared. A paired t-test was used to assess statistically significant differences between preoperative and postoperative outcomes. Subgroup analyses comparing success and failure groups were performed using an independent *t*-test for continuous variables and Chi-Square test for categorical variables. Survival analyses for complete and qualified success were performed using the Kaplan-Meier log-rank test. *P* < 0.05 was considered statistically significant.

## Results

### Study population and baseline characteristics

A total of 49 eyes were included in the analyses. The demographic and baseline characteristics are listed in Table 1. The mean age of the included patients was 65.4 ± 13.7 years. The mean decision IOP and number of preoperative glaucoma medications were 26.9 ± 8.3 mmHg and 3.3 ± 1.0, respectively. The mean preoperative MD was −17.9 ± 9.6 dB, with 37 eyes having an MD < −12 dB, five eyes between −12 and −6 dB, and seven eyes > -6 dB.

**Table 1:** Baseline characteristics of participants undergoing A-stream implantation.

Characteristic	Value
Total participants (*n*)	49
Demographics	
Age, years	65.4 ± 13.7
Left eye, *n* (%)	25 (51.0)
Female, *n* (%)	18 (20)
Diabetes, *n* (%)	11 (22.4)
Hypertension, *n* (%)	11 (22.4)
Baseline IOP and Medications	
Decision IOP (mmHg)	26.9 ± 8.3
Number of glaucoma medications	3.3 ± 1.0
1 medication, *n* (%)	2 (4.1)
2 medications, *n* (%)	5 (10.2)
3 medications, *n* (%)	21 (42.9)
≥4 medications, *n* (%)	21 (42.9)
Glaucoma type	
Primary open angle glaucoma, *n* (%)	28 (57.1)
Uveitic glaucoma, *n* (%)	10 (20.4)
Pigmentary glaucoma, *n* (%)	3 (6.1)
Pseudoexfoliation glaucoma, *n* (%)	6 (12.2)
Steroid induced glaucoma, *n* (%)	2 (4.1)
Preoperative MD	
Overall mean, dB	−17.9 ± 9.6
Mild (MD < −6dB), n (%)	7 (14.3)
Moderate (6 to −12 dB), n (%)	5 (10.2)
Advanced (<−12dB), *n* (%)	37 (75.5)
Previous ocular laser/surgery, *n* (%)	
Cataract surgery	24 (48.9)
Selective laser trabeculoplasty	11 (22.4)
Argon laser trabeculoplasty	2 (4.1)
Trabeculectomy	18 (36.7)
Ahmed valve implantation	1 (2.0)
Penetrating keratoplasty	1 (2.0)
DMEK	1 (2.0)
DSEK	2 (4.1)
Intraoperative MMC (0.4 mg/mL)	
Application time, min	3.1 ± 0.7
<3 min, *n* (%)	9 (18.4)
≥3 min, *n* (%)	40 (81.6)
Combined cataract surgery, *n* (%)	3 (6.1)

*IOP* intraocular pressure, *MD* mean deviation, *DMEK* descemet membrane endothelial keratoplasty, *DSEK* descemet stripping endothelial keratoplasty.

### Primary outcome: surgical success at 6 months

**Fig. 2:**
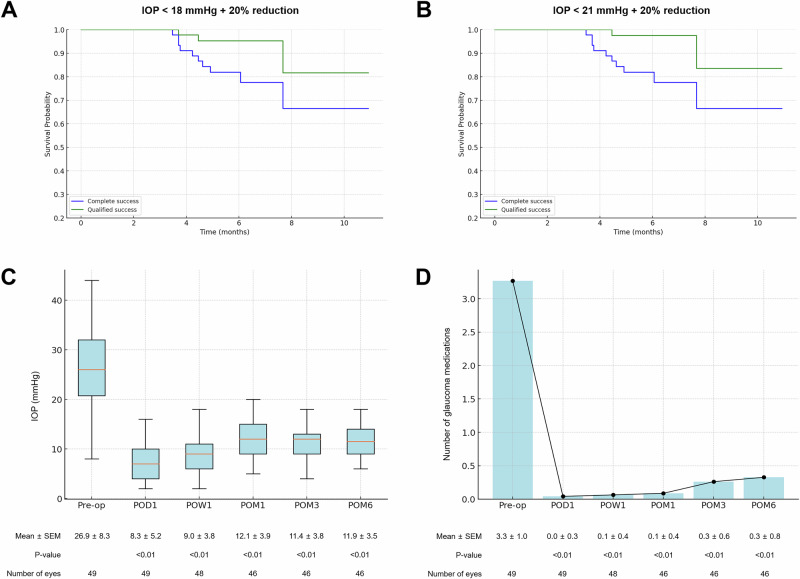
Surgical success, intraocular pressure, and medication reduction following A-stream implantation. **A**, **B** Kaplan-Meier survival curves for surgical success. **A** Success is defined as an intraocular pressure (IOP) ≤ 18 mmHg with at least a 20% reduction from baseline. **B** Success is defined as an IOP ≤ 21 mmHg with at least a 20% reduction from baseline. Complete success (blue) indicates no use of glaucoma medications, while qualified success (green) includes cases requiring medication to achieve target IOP. **C**, **D** Postoperative IOP and glaucoma medication reduction. **C** Box-and-whisker plot showing IOP measured preoperatively (Pre-op) and at postoperative day 1 (POD1), week 1 (POW1), and months 1, 3, and 6 (POM1, POM3, and POM6). Mean IOP ± standard error of the mean (SEM) is indicated below each time point. **D** Bar and line graph illustrating the number of glaucoma medications used preoperatively (Pre-op) and at the same postoperative time points as in (**C**). Mean number of medications ± SEM is indicated below each time point. *P*-values were calculated by comparing preoperative and postoperative values.

At the 6-month follow-up, 77.6% of the eyes achieved complete success and 93.9% achieved qualified success (Table 2, Fig. 2A, B). Notably, these results were consistent even when using an upper IOP cutoff of 21 mmHg; 79.6% and 96.0% achieved complete and qualified success, respectively (Table 2, Fig. 2A, B). No eyes failed because of clinically significant hypotony or the need for reoperation.

**Table 2:** Success rates following A-stream implantation at various postoperative time points.

	Success rate (%)	
	Complete	Qualified
IOP Cut-off 18 mmHg		
Week 1	93.9	98.0
Month 1	87.8	93.9
Month 3	77.6	93.9
Month 6	77.6	93.9
IOP Cut-off 21 mmHg		
Week 1	96.0	100
Month 1	91.8	98.0
Month 3	79.6	96.0
Month 6	79.6	96.0

*IOP* intraocular pressure.

### Comparison of patient characteristics between success and failure groups

To explore the potential differences between the success and failure groups, subgroup analyses were conducted to compare patient characteristics (Supplementary Table 1). Surgical success in this analysis was defined as complete success at the 6-month follow-up, using an IOP threshold of ≤18 mmHg. The failure group was defined as including eyes that did not meet the criteria for complete success, encompassing both qualified success and surgical failure. No significant differences were observed between the groups in terms of age, glaucoma type, combined cataract surgery, intraoperative MMC application time, or preoperative decision IOP. However, patients in the failure group demonstrated a significantly higher IOP at 1 and 3 months postoperatively. In addition, the failure group demonstrated a significantly higher IOP than the success group immediately before ripcord removal (21.1 ± 5.4 mmHg vs. 15.8 ± 5.9 mmHg; *P* = 0.04) and at 1 month after removal (15.6 ± 4.2 mmHg vs. 9.5 ± 2.8 mmHg; *P* < 0.001).

### Changes in IOP and glaucoma medications

At 6 months postoperatively, the mean IOP was 11.9 ± 3.5 mmHg, which demonstrated a significant reduction from the preoperative decision IOP of 26.9 ± 8.3 mmHg (P < 0.01) (Fig. 2C). The mean number of glaucoma medications significantly decreased from 3.3 ± 1.0 preoperatively to 0.3 ± 0.8 at 6 months (*P* < 0.01). Furthermore, 81.6% of the eyes were free of glaucoma medication at 6 months postoperatively (Fig. 2D); 95.9% of the patients used fewer medications than before surgery, and none of the patients required a greater number of medications.

### Effect of adjustable ripcord removal

**Fig. 3:**
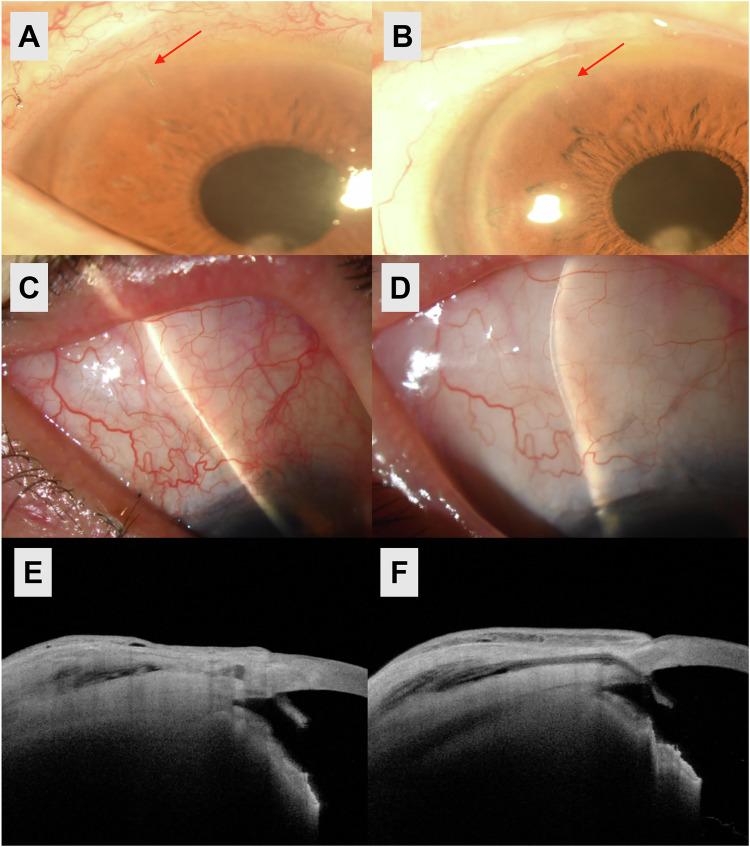
Filtering bleb and anterior segment optical coherence tomography (OCT) images before and after ripcord removal following A-stream implantation. **A**, **C**, **E** Filtering bleb and anterior segment OCT images taken 2 months after A-stream implantation in a 67-year-old man with open-angle glaucoma. The intraocular pressure (IOP) was 20 mmHg without the use of glaucoma medications. The A-stream implant, with the ripcord inside the tube, is visible in the anterior chamber. At this stage, the ripcord was removed to achieve further IOP reduction. **B**, **D**, **F** Images obtained 1 month following ripcord removal, which resulted in IOP reduction to 14 mmHg. The ripcord is no longer visible within the A-stream implant. Along with additional IOP reduction, both the extent and height of the filtering bleb increased. Anterior segment OCT also shows enlargement of the subconjunctival and sub-Tenon fluid spaces surrounding the A-stream implant.

The ripcord was removed at 1.8 ± 1.3 (range 0.0–5.1) months postoperatively in 36 eyes (73.5%). IOP at the time of removal was 18.3 ± 6.6 mmHg, which significantly decreased to 11.5 ± 4.0 at 1-month after ripcord removal (*P* < 0.001). Figure 3 shows representative images of the filtering bleb before and after ripcord removal.

### Complications

Supplementary Table 2 lists the complications observed in this study. Seven cases (14.3%) of transient hypotony with or without choroidal detachment were observed, primarily within the first postoperative week. All seven patients underwent anterior chamber reformation with ophthalmic viscosurgical devices, and the condition resolved in all cases within the first postoperative month without progression to hypotony maculopathy. Notably, no cases of clinically significant hypotony were observed during the follow-up period. Additionally, there were no instances of light perception vision loss, retinal detachment, malignant glaucoma, implant exposure, corneal decompensation, blebitis, or endophthalmitis. Furthermore, a range of potential adverse events reported in previous studies, including device misinsertion into the anterior chamber, misplacement or inadvertent removal of the ripcord, tube lumen blockage by the iris, and cystoid macular oedema, were not observed in this study [15–17].

### Postoperative interventions

A total of 12 interventions were performed (Supplementary Table 2). The most common intervention was anterior chamber reformation (14.3%), followed by needling (6.1%). During the study period, none of the patients required revision or reoperation.

## Discussion

This study evaluated the short-term efficacy and safety of the A-stream, a novel glaucoma shunt incorporating an adjustable ripcord mechanism, during a 6-month follow-up period. The results demonstrated a significant reduction in IOP after A-stream implantation, with high rates of both complete and qualified success. In addition, by enabling stepwise postoperative IOP control through its ripcord mechanism, the A-stream addresses a key limitation of other microshunt devices that lack adjustable IOP control after surgery. These findings suggest that the A-stream could serve as an effective alternative to conventional glaucoma surgeries, offering a promising balance between efficacy and safety.

Glaucoma surgery remains essential for patients who are unresponsive to nonsurgical treatments such as medications and laser therapy [2, 3]. Although the traditional TRAB is effective in lowering IOP, it has significant risks including hypotony and associated complications such as choroidal effusion and maculopathy. Additionally, TRAB has a steep learning curve and requires precise control of scleral flap tension to regulate aqueous humour flow that can lead to variable IOP outcomes. Sudden IOP fluctuations during surgery further increase the risk of severe complications such as suprachoroidal haemorrhage and vision loss [2–4].

MIGS aims to mitigate these issues by providing safer alternatives [5, 18–21]. However, traditional MIGS devices, which primarily target the trabecular meshwork, often have limitations due to pre-existing resistance in the collector and episcleral venous systems. Consequently, MIGS devices typically achieve a less effective IOP reduction than TRAB, limiting overall efficacy in more advanced glaucoma cases [22, 23]. Recently developed microshunt devices such as the PreserFlo offer an attractive solution by creating a reliable outflow pathway via a filtering bleb resembling a TRAB, but with enhanced safety and predictability [13, 14, 24]. A-stream was designed to retain these advantages while maximising efficacy. The A-stream, featuring an internal lumen of 50 µm expandable to 100 µm upon ripcord removal, ensures effective IOP control both in the early and later postoperative stages. This design may offer better IOP control compared to other shunt devices such as the XEN and PreserFlo, which have lumen sizes of 45 µm and 70 µm, respectively [25, 26].

The A-stream demonstrates favourable IOP reductions comparable to those of established devices and conventional glaucoma surgeries. Our 6-month follow-up results showed a mean IOP reduction from 26.9 ± 8.3 mmHg to 11.9 ± 3.5 mmHg, with complete success in 77.6% and qualified success in 93.9% of eyes. These findings align well with previously published outcomes for other MIGS devices, although differences in follow-up durations and patient populations preclude direct comparisons. For example, XEN, another glaucoma shunt device, has demonstrated IOP reductions from 20–25 mmHg to the low to mid-teens at 1–2 years postoperatively, with complete success rates of 33.8–78% and qualified success rates of 67.6–78% [20, 26–28]. Similarly, the PreserFlo has shown mean IOP reductions to 14.7 ± 0.6 mmHg, with complete and qualified success rates of 51.9–76.9% and 68.3–92.5%, respectively, at 1 year [15, 20, 21]. In a primary tube versus TRAB study, augmented TRAB reduced IOP from a mean of 23.3 mmHg to 13.8 mmHg, and Baerveldt tube implantation reduced IOP from 23.9 mmHg to 12.4 mmHg [29, 30]. These findings suggest that the A-stream may achieve IOP reductions comparable to those observed with traditional TRAB and tube-shunt surgeries, which are highly effective in reducing IOP.

In this study, ripcord removal at an average of 1.8 ± 1.3 months postoperatively led to a significant decrease in IOP from 18.3 ± 6.6 mmHg to 11.5 ± 4.0 mmHg. To optimise outcomes, specific criteria for ripcord removal were applied, including IOP > 16 mmHg and removal typically performed ~2 months after surgery, with exceptions made based on individual IOP levels and bleb morphology. This approach was designed to prevent unnecessary hypotony caused by premature ripcord removal while ensuring adequate IOP control. Additionally, IOP was monitored 30 min after ripcord removal to detect immediate hypotony or related complications, but notably, no cases of IOP < 6 mmHg were observed following removal. This technique provides an alternative to laser suture lysis in TRAB, offering potentially more predictable outcomes with fewer complications. Further studies are warranted to evaluate its long-term efficacy and refine removal protocols for optimal outcomes.

Similarly, studies on stented PreserFlo have demonstrated the effectiveness of intraluminal ripcords in reducing early postoperative hypotony and achieving stable IOP following ripcord removal [31–33]. These findings align with the observed benefits of the integrated ripcord mechanism of the A-stream. However, differences in device design, such as the A-stream’s larger lumen diameter (100 µm vs. 70 µm in PreserFlo) and shorter tube length, may influence long-term IOP control and surgical outcomes, warranting further investigation [34–36].

In cases where low IOP persisted beyond 2–3 months, the ripcord was retained for an extended period, which occasionally resulted in the ripcord becoming completely buried under the conjunctiva or Tenon’s capsule, requiring a small incision for removal. Alternatively, if the ripcord remained exposed above the conjunctiva but removal was unnecessary due to sustained low IOP, it could be trimmed with scissors under a slit lamp and buried beneath the conjunctiva to prevent foreign body sensation or infection. Therefore, the timing of ripcord removal must be carefully determined, considering IOP levels, elapsed time after surgery, and wound healing processes in the filtering bleb. Additional studies are necessary to gather more data and refine the decision-making process for the optimal timing of ripcord removal.

Another important role of the ripcord is mitigating the risk of serious hypotony, particularly during the early postoperative period. We observed a transient hypotony rate of 14.3%, which is similar to the rates reported for other microshunt devices, which range from 10% to 40% [7, 23, 26, 37]. However, despite the presence of a ripcord, the rate was higher than expected. This unexpected outcome may be due to the larger 100 µm lumen diameter of the A-stream. Nonetheless, the majority of hypotony cases are resolved within 1 month without severe complications such as hypotony maculopathy. Although anterior chamber reformation was sometimes performed proactively to prevent complications, its necessity in all cases remains uncertain, as some instances of transient hypotony may have resolved spontaneously. The current lumen and ripcord configuration was selected through extensive experimental testing and reflects a careful balance between preventing hypotony and maintaining sufficient aqueous outflow for effective IOP control [13, 14]. Given these considerations, close monitoring and timely intervention are essential during the early postoperative period to manage potential complications.

In this study, three patients underwent postoperative needling due to inadequate IOP control despite ripcord removal and the use of anti-glaucoma medications. Importantly, needling was not performed to address tube obstruction as the tube opening remained patent. Instead, the needling procedure was performed in order to target bleb encapsulation and fibrosis, a limitation commonly observed in glaucoma filtering surgeries, including minimally invasive bleb surgeries and conventional TRAB [38–41]. Although needling may release encapsulated bleb effectively, its long-term efficacy following A-stream implantation remains uncertain due to the risk of fibrosis recurrence [9]. In cases in which the IOP remains uncontrolled despite ripcord removal, further studies are needed to evaluate the effectiveness of needling and explore alternative strategies for long-term management of such cases.

The A-stream was designed to be placed under a scleral flap, which distinguishes it from other microshunt devices that are inserted directly into the anterior chamber through the sclera. This approach is intended to prevent implant exposure, a complication that has been reported with other tube devices of similar diameters [16, 17, 42–44]. Additionally, placement under the scleral flap creates a space between the tube and Tenon’s capsule, which potentially reduces the risks of compression or occlusion by the Tenon’s capsule [42–45]. Particular attention was paid to position the wings of the A-stream under the scleral flap during implantation. The wings serve as fixation points, providing stability during and after surgery. Positioning the wings under the scleral flap minimises direct contact with the overlying conjunctiva or Tenon’s capsule, thereby reducing the risk of tissue erosion. In this study, no cases of tube migration or exposure were observed during the 6-month follow-up period. The long-term safety and performance of the A-stream design and positioning require further evaluation to confirm its efficacy and stability over time.

This study has several limitations. First, it was limited by its retrospective design, small sample size, and lack of a control group, which precludes direct comparison with other devices. Future prospective randomised controlled trials are required to compare the outcomes of the A-stream with those of established devices. Second, the small sample size limited the statistical power to identify the variables influencing surgical outcomes. Larger studies using multivariate approaches are necessary to better understand the factors influencing success rates. Third, pre- and postoperative endothelial cell counts were not compared. However, given that the A-stream is made of medical-grade silicone and has a smaller luminal diameter than other glaucoma drainage devices, it is expected to cause comparable or even less endothelial damage. Neither endothelial decompensation nor corneal oedema was reported in this study, although further confirmation is required. Finally, the study included primary open-angle glaucoma cases, several secondary glaucoma cases, and cases with a history of failed initial filtering surgery. This heterogeneity made it challenging to accurately assess the primary efficacy of A-stream. Future studies should include prospective designs with larger sample sizes and longer follow-up periods to validate these findings and provide more comprehensive assessments of the long-term safety and efficacy of the A-stream glaucoma shunt.

In conclusion, the A-stream glaucoma shunt was a safe and effective surgical option for patients with glaucoma, which demonstrated excellent short-term outcomes. The ripcord prevented significant postoperative hypotony and enabled stepwise IOP reduction in a stable, effective manner. Further studies are required to elucidate the long-term outcomes of A-stream implantation and compare them with other conventional glaucoma surgeries to fully establish its efficacy and safety profile.

Supplemental material is available at Eye’s website.

## Summary

### What was known before

- Trabeculectomy (TRAB) remains the gold standard for glaucoma surgery but carries significant risks, including hypotony and unpredictable outcomes.
- Minimally invasive glaucoma surgery (MIGS) devices, though safer, often exhibit suboptimal IOP reduction in advanced glaucoma.
- Recent tube shunt devices, such as XEN and Preserflo, provide more consistent IOP control but lack mechanisms for postoperative adjustment.

### What this study adds

- The A-stream glaucoma shunt, with its adjustable ripcord mechanism, allows for stepwise IOP control postoperatively, overcoming a key limitation of existing tube shunt devices.
- A-stream demonstrated significant IOP reduction and high success rates over a 6-month period, with a favourable safety profile, showing promise as an alternative to conventional glaucoma surgeries.

## Supplementary information

Supplementary Table 1

Supplementary Table 2
