# Supplementary material for: Short-term efficacy and safety of A-stream glaucoma shunt: a 6-month study
Source: Eye (Lond). 2025 Feb 21;39(8):1584–91. doi: 10.1038/s41433-025-03728-y (PMC12089429; doi:10.1038/s41433-025-03728-y)
Supplement: Supplementary file 1 — Supplementary Table 1 [file 41433_2025_3728_MOESM1_ESM.docx]

Supplementary Table 1. Comparison of Patient Characteristics Between Success and Failure Groups.

| Characteristic | Success  (n = 38) |  | Failure  (n = 11) |  | *p-value* |
| --- | --- | --- | --- | --- | --- |
| Age (years) | 62.9 ± 13.8 |  | 58.3 ± 22.1 |  | *0.45* |
| Sex (n, M:F) | 27:11 |  | 4:7 |  | 0.04* |
| Preoperative MD (dB) | -17.8 ± 9.82 |  | -17.7 ± 9.6 |  | *0.99* |
| CCT (µm) | 543.7 ± 68.8 |  | 556.1 ± 80.6 |  | *0.68* |
| Diagnosis (POAG:non-POAG) | 20:18 |  | 8:3 |  | *0.13** |
| POAG (n,%) | 20 (52.6) |  | 8 (72.7) |  |  |
| Uveitic glaucoma (n,%) | 7 (18.4) |  | 3 (27.3) |  |  |
| Pigmentary glaucoma (n,%) | 3 (7.9) |  | 0 (0) |  |  |
| Pseudoexfoliation glaucoma (n,%) | 6 (15.8) |  | 0 (0) |  |  |
| Steroid induced glaucoma (n,%) | 2 (5.2) |  | 0 (0) |  |  |
| Previous ocular laser/surgery |  |  |  |  |  |
| Cataract surgery (n, Yes:No) | 18:20 |  | 6:5 |  | *0.7** |
| Laser trabeculoplasty (n, Yes:No) | 11:27 |  | 2:9 |  | *0.5** |
| Trabeculectomy (n, Yes:No) | 12:26 |  | 7:4 |  | *0.09** |
| Keratoplasty (n, Yes:No) | 3:35 |  | 1:10 |  | *0.9** |
| Combined cataract surgery (n, Yes:No) | 3:35 |  | 0:11 |  | *1.0** |
| Intraoperative MMC 0.4 mg/mL (min) | 3.0 ± 0.6 |  | 3.3 ± 1.2 |  | *0.49* |
| Intraoperative MMC 0.4 mg/mL |  |  |  |  |  |
| < 3 min (n, %) | 6 (15.8) |  | 3 (27.3) |  | *0.2** |
| ≥ 3 min (n, %) | 32 (84.2) |  | 8 (72.7) |  |  |
| IOP (mmHg) |  |  |  |  |  |
| Decision IOP | 28.8 ± 10.8 |  | 26.8 ± 6.1 |  | *0.62* |
| POM1 | 11.6 ± 4.2 |  | 15.9 ± 5.5 |  | *0.02* |
| POM3 | 10.4 ± 3.7 |  | 17.3 ± 5.6 |  | <0.001 |
| Number of medications used |  |  |  |  |  |
| Preoperative | 3.7 ± 0.8 |  | 3.6 ± 0.7 |  | *0.87* |
| POM1 | 0.0 ± 0.0 |  | 0.9 ± 1.3 |  | *0.09* |
| POM3 | 0.1 ± 0.2 |  | 0.8 ± 1.2 |  | *0.14* |
| Ripcord removal |  |  |  |  |  |
| Removed (n,%) | 26 (68.4) |  | 10 (90.9) |  | *0.36** |
| Ripcord removal time (months) | 3.1 ± 2.8 |  | 1.4 ± 1.1 |  | *0.12* |
| IOP before removal (mmHg) | 15.8 ± 5.9 |  | 21.1 ± 5.4 |  | *0.04* |
| IOP at 1-month after removal (mmHg) | 9.5 ± 2.8 |  | 15.6 ± 4.2 |  | <0.001 |
|  |  |  |  |  |  |

M, male; F, female; CCT, central corneal thickness; POAG, primary open-angle glaucoma; MMC, mitomycin-C; IOP, intraocular pressure; POM, postoperative month

Surgical success was defined as achieving an IOP ≤ 18 mmHg with at least a 20% IOP reduction from baseline, without the use of anti-glaucoma medications, at the 6-month postoperative follow-up. Only complete success is included in this table.

The failure group was defined as including eyes that did not meet the criteria for complete success, encompassing both qualified success and surgical failure.

Data are presented as mean ± standard deviation or number of patients (%), unless otherwise indicated.

*Data were analyzed using the chi-square test; otherwise, they were analyzed using the independent t-test.
