# Supplementary material for: Short-term efficacy and safety of A-stream glaucoma shunt: a 6-month study
Source: Eye (Lond). 2025 Feb 21;39(8):1584–91. doi: 10.1038/s41433-025-03728-y (PMC12089429; doi:10.1038/s41433-025-03728-y)
Supplement: Supplementary file 2 — Supplementary Table 2 [file 41433_2025_3728_MOESM2_ESM.docx]

**Supplementary table 2. Postoperative complications and interventions following A-stream implantation**

|  | **n (%)** | |
| --- | --- | --- |
| **Complications** | **Early (< 3 months)** | **Late (> 3 months)** |
| Clinically significant hypotony | 0 (0) | 0 (0) |
| Transient hypotony | 7 (14.3) | 0 (0) |
| Choroidal detachment | 4 (8.2) | 0 (0) |
| Hypotony maculopathy | 0 (0) | 0 (0) |
| Leak/dehiscence | 2 (4.1) | 0 (0) |
| Hyphema | 0 (0) | 0 (0) |
| Implant exposure | 0 (0) | 0 (0) |
| Serious complications* | 0 (0) | 0 (0) |
| * Serious complications include vitreous hemorrhage, retinal detachment, suprachoroidal hemorrhage, malignant glaucoma, endophthalmitis or blebitis, corneal decompensation, and light perception loss. | | |
| **Interventions** | **n (%)** | |
| Needling | 3 (6.1) | |
| Anterior chamber reformation | 7 (14.3) | |
| Anterior chamber tapping | 0 (0) | |
| Conjunctival suture | 2 (4.1) | |
